# Identifying genetic risk loci for diabetic complications and showing evidence for heterogeneity of type 1 diabetes based on complications risk

**DOI:** 10.1371/journal.pone.0192696

**Published:** 2018-02-14

**Authors:** Nandita Mukhopadhyay, Janelle A. Noble, Manika Govil, Mary L. Marazita, David A. Greenberg

**Affiliations:** 1 Center for Craniofacial and Dental Genetics, Department of Oral Biology, School of Dental Medicine, University of Pittsburgh, Pittsburgh, Pennsylvania, United States of America; 2 Children’s Hospital Oakland Research Institute, Oakland, California, United States of America; 3 Department of Human Genetics, Graduate School of Public Health, University of Pittsburgh, Pittsburgh, Pennsylvania, United States of America; 4 Clinical and Translational Science Institute, School of Medicine, University of Pittsburgh, Pittsburgh, Pennsylvania, United States of America; 5 Battelle Center for Mathematical Medicine, Nationwide Children’s Hospital, Columbus, Ohio, United States of America; 6 Department of Pediatrics, Wexner Medical Center, Ohio State University, Columbus, Ohio, United States of America; University of Alabama at Birmingham, UNITED STATES

## Abstract

There is a growing body of evidence suggesting that type 1 diabetes (T1D) is a genetically heterogeneous disease. However, the extent of this heterogeneity, and what observations may distinguish different forms, is unclear. One indicator may be T1D-related microvascular complications (MVCs), which are familial, but occur in some families, and not others. We tested the hypothesis that T1D plus MVC is genetically distinct from T1D without MCV. We studied 415 families (2,462 individuals, 896 with T1D) using genome-wide linkage analysis, comparing families with and without MVC. We also tested for interaction between identified loci and alleles at the *HLA-DRB1* locus. We found significant linkage scores at 1p36.12, 1q32.1, 8q21.3, 12p11.21 and 22q11.21. In all regions except 1p36.12, linkage scores differed between MVC-based phenotype groups, suggesting that families with MVCs express different genetic influences than those without. Our linkage results also suggested gene-gene interaction between the above putative loci and the HLA region; HLA-based strata produced significantly increased linkage scores in some strata, but not others within a phenotype group. We conclude that families with type 1 diabetes plus MVCs are genetically different from those with diabetes alone.

## Introduction

Much of the morbidity and mortality of type 1 diabetes (T1D, insulin-dependent diabetes) results from the microvascular complications (MVC) that are caused, in part, by high blood glucose levels. These MVCs, also seen in type 2 diabetes (T2D, non-insulin-dependent diabetes), include: nephropathy, which can cause kidney disease leading to dialysis, kidney transplant, and even death; retinopathy, which can lead to blindness; and neuropathy, which can lead to loss of limbs. MVCs of T1D are triggered by high blood glucose levels; however, some T1D families are observed to express MVCs while others do not [1, 2], suggesting a strong genetic component in MVC expression. The presence of T1D MVCs in some patients and families but not others also implies heterogeneity within T1D itself, but the extent of that heterogeneity, and whether these different phenotypic forms are correlated with the presence of MVCs, is currently unclear.

Diabetes (high blood sugar) has long been recognized to be a heterogeneous disease, but, originally, even insulin-dependent and non-insulin dependent diabetes were not differentiated. The early discovery of a strong association of HLA with T1D, and a lack of HLA association with T2D, demonstrated that the two forms of diabetes differ genetically, further supported by the eventual discovery of differences in the mechanism that produces high blood sugar in T1D versus T2D. Since then, several other forms of diabetes have been identified, including neonatal diabetes mellitus and maturity onset diabetes of the young (MODY), both of which are genetically distinct from other forms of diabetes.

To our knowledge, genetic heterogeneity within T1D itself has not been a subject of recent concerted research, although recently Liley et al. (2017) developed an algorithm to test whether different phenotypic groups represented different genetic architectures. They applied this method to T1D antibody data examining, among other tests, whether differing autoantibody positivity represented possible different subgroups of T1D. Their findings, like our own, further strengthen the hypothesis that there is heterogeneity within the T1D phenotype.

Studies have also shown a strong genetic influence on MVCs [1, 2] but few studies have asked the question whether the combination of T1D plus T1D-related MVCs represents a distinct form of T1D, or if MVCs are influenced by genes that are different from those that influence T1D itself. Either hypothesis is consistent with the observation that MVCs are not seen in every T1D patient or in every family of such patients. Given the genetic complexity of T1D, the apparent familial component in the expression of MVCs, and the variety of symptoms that manifest in T1D and MVCs, it is logical to conclude that both hypotheses are true, i.e., there is variation among the genes that are involved in T1D as well as in T1D-related MVCs, a conclusion supported by the results of our previous work [3, 4].

In our previous work, we showed that the risk for MVCs is influenced by specific HLA alleles, particularly at the *DRB1* locus [3]. In follow-up studies, we sought to identify loci on chromosome 6 that are genetically linked to the expression of MVCs, and to identify epistatic interaction between these loci and the HLA region [4]. In that work, we stratified T1D family data on both the *HLA-DRB1* type of the T1D proband and on the particular type(s) of MVC that were diagnosed in that proband. That analysis was structured in such a fashion that multiplex T1D case-containing families in which the proband had at least one MVC (classified as “affected”) were contrasted against multiplex T1D families without MVC (classified as “unaffected”) in any T1D-expressing family member (those family members without T1D did not contribute to the results). Using linkage analysis, we discovered evidence for two loci on chromosome 6, outside the HLA region, linked to MVCs. We found that one of the loci influenced primarily the expression of retinopathy while the other appeared to affect nephropathy expression. We stratified the linkage further on specific *HLA-DRB1* alleles in order to detect evidence of interaction between those loci and HLA, using a technique developed by us for this purpose [5, 6]. We found that whether the *HLA-DRB1* allele present in the proband was DRB1*03:*01* or DRB1*04:*01* differentially affected MVC expression, suggesting that the different HLA alleles interacted with the newly identified loci. We emphasize that that previous work examined only chromosome 6.

**Fig 1:**
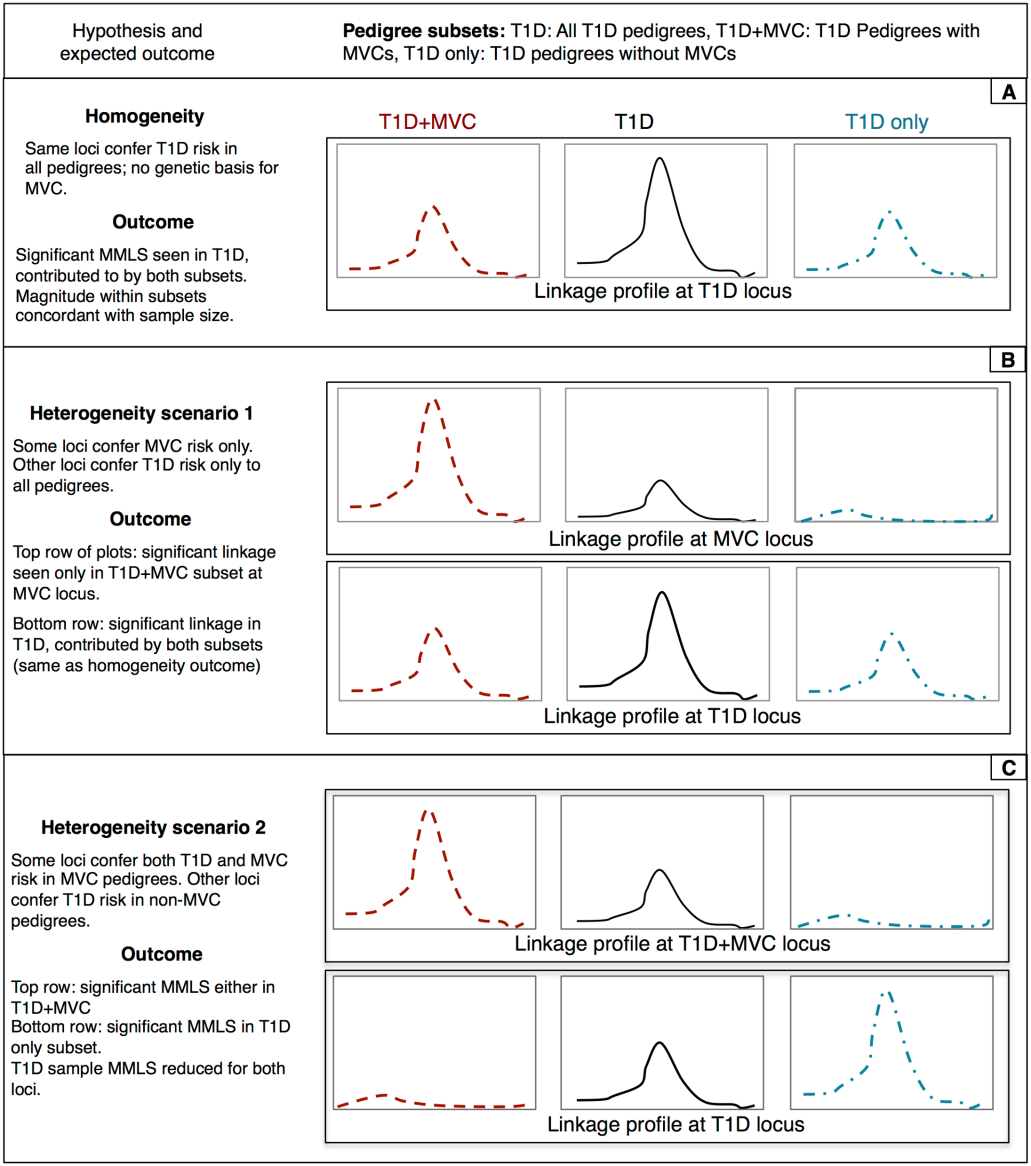
Hypothetical linkage score profiles expected under (A) absence of genetic heterogeneity, (B) presence of heterogeneity due to additional loci conferring MVC risk only, and (C) presence of heterogeneity due to different T1D risk conferring loci MVC vs. non-MVC pedigrees.

In the current work, we have carried out a more extensive investigation of the overarching question: are T1D families in which there are MVCs genetically different from T1D families in which such MVCs do not occur? In this study, the search for T1D- and MVC-influencing susceptibility loci has been expanded beyond chromosome 6 to the rest of the genome. As in our previous work [4], our analytical approach consists of performing linkage analyses on two groups of families, those with, and those without MVCs (in addition to all T1D families), testing whether loci are detected in one group as opposed to the other. Fig 1 lists the different hypotheses tested, along with illustrative linkage profiles that we would expect to see for each of the hypotheses, namely, homogeneity (panel A), heterogeneity due to separate T1D and MVC loci (panel B) and heterogeneity arising from differences in T1D risk-conferring loci (panel C). In addition to such differences, we also ask if there is evidence of interaction between HLA and putative loci outside of chromosome 6 that are linked to T1D and MVCs.

### Terminology

The following labels have been used to denote subsets of pedigrees throughout this report. Labels used for phenotypic subgrouping are: **T1D** − all pedigrees with one or more T1D affected members (with or without complications); **Complications** − subset of T1D pedigrees containing members affected with T1D as well as affected with one or more MVCs (nephropathy, retinopathy and neuropathy); **No complications** − T1D pedigrees without MVCs. Phenotypic subgroups are further stratified on the basis of their probands’ *HLA-DRB1* genotypes as follows: 3/*, 4/* − genotypes contain at least one copy of the DRB1*03:*01* or DRB1*04:*01* allele, respectively; **3/{3,X}**–subjects with at least one copy of the DRB1*03:*01* allele and zero copies the DRB1*04:*01* allele; **4/{4, X}** − subjects with at least one copy of the DRB1*04:*01* allele and zero copies of the DRB1*03:*01* allele; **3/4** –subjects with one copy of the DRB1*03:*01* and one copy of the DRB1*04:*01* allele; and **X/X** − subjects with neither the DRB1*03:*01* allele, nor the DRB1*04:*01* allele. Results for HLA unstratified phenotypic groups are labeled as such, e.g. “unstratified **T1D**” refers to the set of pedigrees, not stratified on HLA type, analyzed such that anyone with the T1D phenotype, irrespective of MVC status, is designated “affected”. Phenotype subgroups and their *HLA-DRB1* strata assignment are discussed in further detail in the Methods section.

**MMLS**: MMLS refers to the maximized maximum LOD score, which we used to measure linkage in our study.

## Results

### Sample size

**Fig 2:**
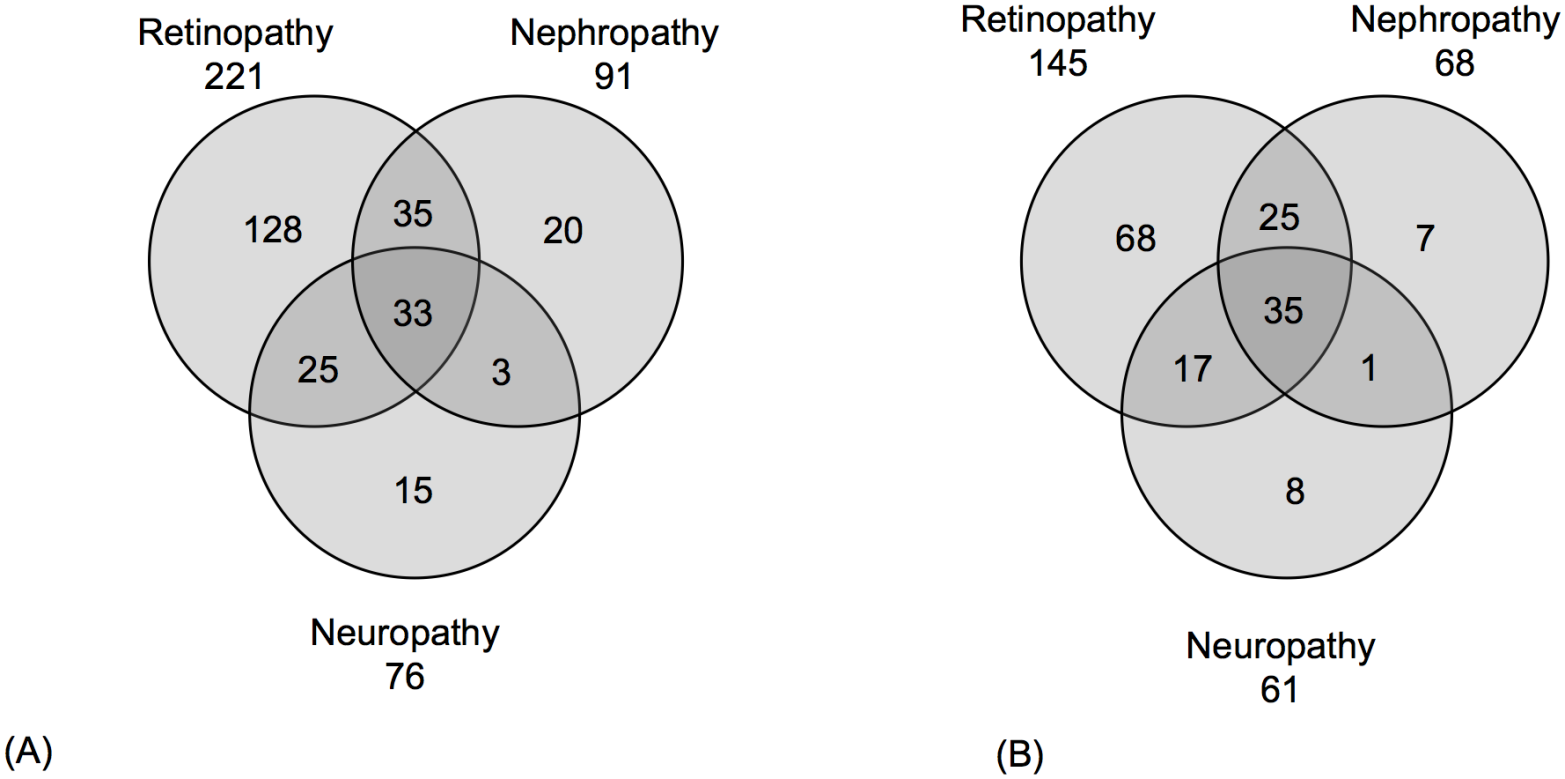
Counts of (A) subjects and (B) pedigrees by specific MVCs, retinopathy, nephropathy, and neuropathy showing overlap across the three categories.

Table 1 shows the counts of subjects within the three phenotype groups analyzed in this study. Pedigrees with T1D+ MVCs constitute a smaller subset than those with T1D only. Fig 2 contains a breakdown of subjects by the type of MVC (namely Retinopathy, Nephropathy and Neuropathy). Fig 2 shows that in our study sample, not only does a large proportion of the individuals develop more than one MVC, but also that multiple MVCs cluster within pedigrees, underscoring the need for to combine all three MVCs into a single phenotype for linkage analysis.

**Table 1:** Sample sizes for phenotype groups.

Phenotype group	Pedigrees*	Individuals (Male, Female)	Affected	Unaffected	Genotyped
**T1D**	415	2462 (1259, 1203)	896	1550	1995
**Complications**	154	975 (483, 492)	232	111	761
**No Complications**	259	1478 (770, 708)	538	930	1225

*Pedigrees with at least 2 phenotyped and 2 genotyped individuals are informative for linkage. Note: the counts of affected and unaffected samples (columns 4 and 5) are determined as described in the “Definition of phenotypes based on T1D and MVC” section.

### Genome-wide linkage

**Fig 3:**
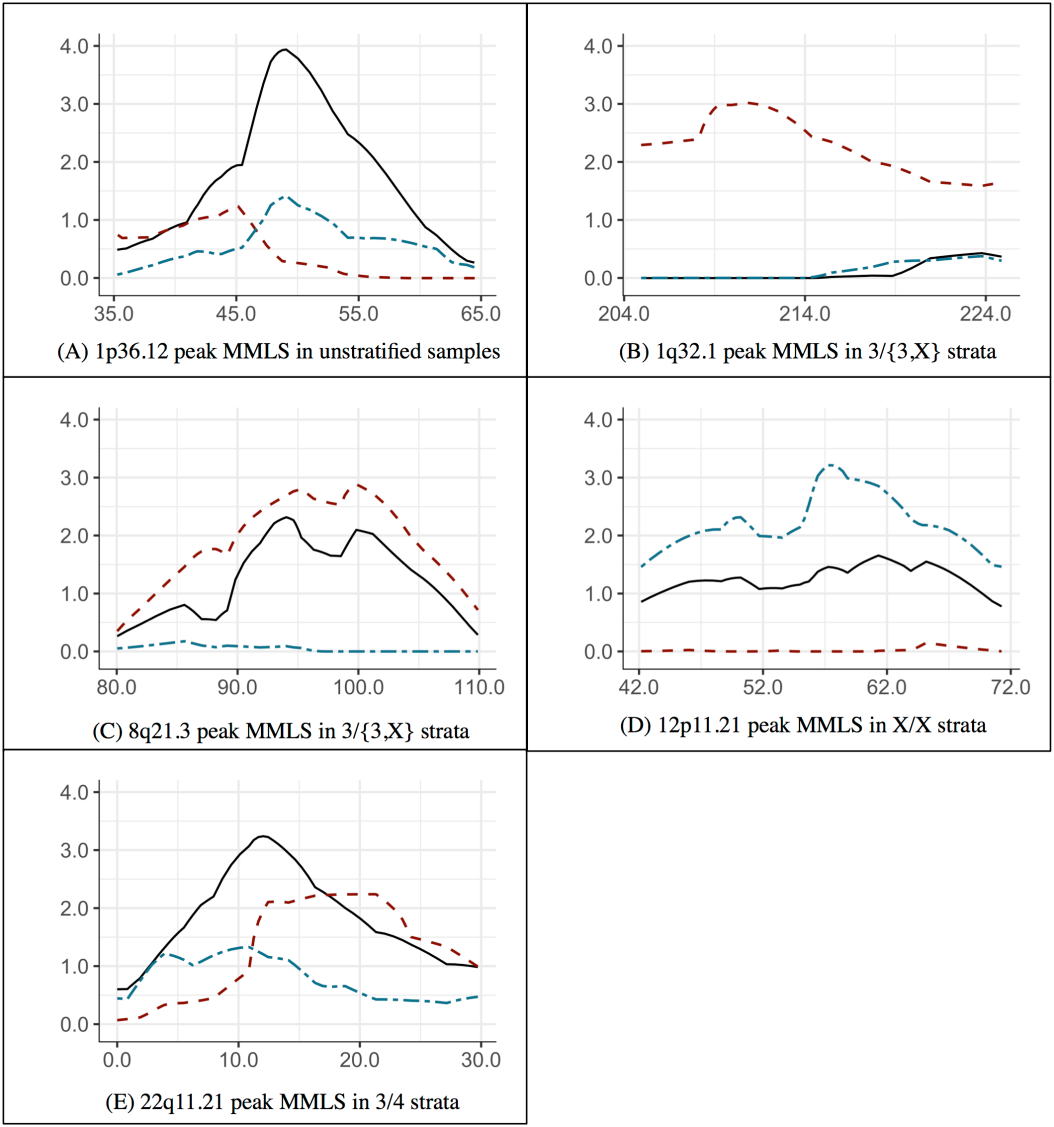
MMLS of T1D, Complications and No complications phenotype groups. Note: In each plot, solid lines represent T1D, dashed line Complications, and dotted lines No Complications; x-axis represents genetic position on the chromosome in cM; y-axis represents MMLS score. Each plot shows a region spanning approximately 30 cM around the peak position.

Several linkage regions with significant or near-significant MMLS scores were observed for the three phenotypic groups. In some cases the high scores were seen only in groups of families stratified on specific *HLA*-*DRB1* alleles. Table 2 and Fig 3 contain linkage results for the specific *HLA-DRB1* stratum in which the significant (or near-significant) linkage peak was observed. All three phenotypic categories are listed. Table 2 shows the positions, highest MMLS, LODs, and estimated proportion of linked pedigrees in those categories. Also shown are the pre-divided sample test chi-square statistics and *p*-values calculated from the reported MMLSs for **Complications, No complications** (sub-samples) and **T1D** (all families, regardless of MVC status). This test is used to detect locus heterogeneity between groups [7]. The S1 Table (supporting material) contains MMLSs in linkage regions for all strata of the phenotype groups.

**Table 2:** Regions with significant linkage MMLS.

Position (cM)	Phenotype; *DRB1* Stratum	MMLS	LOD	α (%)	p-value
**Chromosome 1p36.12**					
49	Unstratified T1D	**3.94**	-90	17	NA
45	Unstratified Complications	1.23	-9.82	26	
49	Unstratified No complications	1.42	-59.31	13	
**Chromosome 1q32.1**					
215	T1D: 3/{3,X}	0.00	-54	0	14.09; 2.0e-4
210	Complications: 3/{3,X}	**3.02**	2.79	89	
215	No complications: 3/{3,X}	0.04	-32	4	
**Chromosome 8q21.3**					
94	T1D: 3/{3,X}	2.31	-35.81	16	3.04 (0.08)
100	Complications: 3/{3,X}	**2.88**	2.02	64	
94	No complications: 3/{3,X}	0.09	-14	10	
**Chromosome 12p11.21**					
61	T1D:X/X	1.66	-11	26	7.23 (0.007)
54	Complications: X/X	0.02	-1.54	13	
57	No complications: X/X	**3.21**	-0.09	52	
**Chromosome 22q11.21**					
12	T1D:3/4	**3.24**	-1.39	56	1.43 (0.23)
17	Complications:3/4	2.22	2.22	100	
11	No complications:3/4	1.33	-4.00	43	

Maximum MMLS, LOD, and estimated linked percentage of pedigrees (α) observed for the three phenotypes, T1D, Complications and No complications, only for the *DRB1* allele strata corresponding to each linkage peak above 3.0. The last column contains the significance p-value of the pre-divided sample test.

### Chromosome 1p36.12 peak region

A significant MMLS peak (3.94, 49 cM) is observed in unstratified **T1D** (i.e., the entire sample); this region has not been previously reported in connection with T1D. Much of the signal appears to originate in the DRB1*03:*01* carriers, i.e. the 3/* stratum (3.27, 50 cM) (Fig 3A and the “Chromosome 1p 36.12” part of the table in S1 Table). MMLS of the other **T1D** strata at this location are below the significance threshold (Fig 3A). The unstratified **T1D** MMLS is the highest observed across all phenotype groups and *DRB1* strata in our analyses. MMLSs in the other groups and other strata are well below the suggestive threshold (part “Chromosome 1p 36.12” of table in S1 Table). We conclude that this region contains a gene that increases the risk of T1D, and appears to interact with the HLA region. Both the **Complications** and the **No complications** groups appear to be influenced by this T1D locus in proportion to their representation in the T1D sample, and therefore, the presence or absence of MVCs alone does not account for heterogeneity at this locus. The pre-divided sample test χ^2^ statistic cannot be calculated using the equation in Section 2.6, as the total of the pre-divided sample MMLSs is below the undivided sample’s MMLS.

### Chromosome 1q32.1 peak region

At this location, the **Complications** sample’s **3/{3,X}** stratum shows a significant MMLS peak, along with a correspondingly notable LOD score (2.79) and high α (% families showing linkage) value estimates (89%). Neither the **No complications** group nor the **T1D** group shows any evidence for a locus at this location (Fig 3B), supporting the existence of a locus contributing susceptibility to MVCs, but not T1D itself. This locus appears to interact with the *HLA-DRB1* gene, and specifically the 03:01 allele, as strata other than **3/{3,X}** of the **Complications** group produce weak or no evidence of a linked locus. This peak spans the *IL10* gene, previously reported as being associated with T1D and kidney disease. The pre-divided sample test statistic is significant, the strong linkage signal coming from the **Complications** subgroup (Table 2), thereby providing evidence strongly in favor of genetic heterogeneity between families with/without MVCs at this locus. Reduction in heterogeneity within T1D is also indicated since the **Complications** group becomes nearly homogeneous (α = 89%) at this locus.

### Chromosome 8q21.3 peak region

The **Complications** phenotype result shows a near-significant peak (MMLS = 2.88) at this location, and a suggestive LOD score (2.02) only within the **3/{3,X}** stratum. The corresponding MMLS score in this stratum is 2.31, while the **No complications** group’s **3/{3,X}** stratum shows no evidence for linkage. The MMLS scans of both the **T1D** *and* **Complications** groups show both profile and magnitude similarities (Fig 3C). This suggests that much of the linkage signal at this location can be attributed to pedigrees with MVCs, although the pre-divided sample test does not reach significance. Thus, this locus appears to contain a T1D risk gene that may also predisposes to MVCs and in addition interacts with HLA. As we exclude first the T1D-only families, then non-3/* families, and finally the non-**3/{3,X}** families, both the MMLS and LOD scores increase, despite the reduction in sample size due to each exclusion. The strata-specific MMLSs are listed in the “Chromosome 8q21.3” part of the table in S1 Table).

### Chromosome 12p11.21 peak region

On chromosome 12p11.21, no stratum of the **T1D** group produces any evidence for a linked locus and all strata of the **Complications** group have MMLS scores close to 0. However, the **No complications X/X** stratum has a significant MMLS (3.21), which is the strongest signal observed genome-wide for the **No complications** phenotype. The **No complications** group as a whole, and all strata of that group, except, **X/X** show no evidence for a locus. Fig 3D shows MMLS curves spanning this region of positive linkage for the **X/X** strata of **T1D**, **Complications** and **No complications** groups. These results provide support for a T1D-related locus at **12p11.21** that appears to be *protective* against MVCs, the degree of protection further altered by the HLA genotype. The pre-divided sample test statistic is highly significant, supporting the hypothesis that the **Complications** and **No complications** groups are genetically different from each other at that locus.

### Chromosome 22q11.21 peak region

A significant peak (MMLS 3.24) is observed at 22q11.21 in the **3/4** stratum of the **T1D** group. Neither the **T1D** group as a whole nor other **T1D** strata, show evidence for linkage. The total evidence for linkage in the two subgroups of the **T1D** group, **Complications** and **No complications**, sums approximately to the signal in the T1D group (Fig 3E). It is likely that there is a T1D risk gene in this region, and the presence of both **3** and **4** alleles increases this risk compared to the other *DRB1* genotypes. The linkage evidence also suggests that strong gene-gene interaction between *DRB1* and this locus maybe the basis for heterogeneity observed among the T1D pedigrees, rather than presence or absence of MVCs. Nonetheless, it is interesting that the **Complications** group showed evidence of linkage and no evidence of heterogeneity (identical MMLS and LOD scores), indicating an increased risk for MVCs in the **3/4** pedigrees.

## Discussion

The primary aim of this study was to test the hypothesis that T1D families with MVCs are genetically different from T1D families without MVCs, a topic that has not been adequately explored to date. Here, our approach for detecting heterogeneity in T1D families by conditioning on phenotypic status as well as *HLA-DRB1* genotypes [3, 4] was expanded to the entire genome. To our knowledge, this is the first such genome-wide study to analytically explore the genetic heterogeneity of T1D based on MVCs.

Our analyses found conclusive evidence that families with MVCs are indeed genetically different from those without MVCs. We also found strong indications of epistatic gene-gene interaction between alleles at the *HLA-DRB1* locus and genes located on other chromosomes. In addition, we identified novel genomic regions linked to both T1D and MVCs.

### Genetic heterogeneity based on presence or absence of MVCs

Our (not mutually exclusive) hypotheses were: (a) T1D risk-conferring genes differ between families affected with T1D only versus those also affected with MVCs, and (b) T1D risk genes do not differ, but additional loci confer risk of an MVC. To test hypothesis (a), subsets of T1D pedigrees were created based on presence or absence of MVCs, with the expectation that “purer” or more *phenotypically* homogenous subsets (although smaller in size) would result in increased linkage signals, reflecting *genetic* homogeneity. Within the **Complications** pedigrees, the definition of affectedness was the presence of both T1D and MVC, allowing us to test hypothesis (b).

We found linkage regions contributing to MVC risk specifically, as well as showing *altered* T1D risk in phenotypic subgroups as compared to the full T1D sample. The 1q32.1 peak suggests there is a genotype at that locus conferring increased risk for MVCs and, correspondingly, the lack of that genotype leads to protection against MVCs. We demonstrated this T1D-risk effect by showing that markers at that chromosomal locus co-segregated with MVCs but not with T1D itself, neither in the **T1D** nor in the **No complications** groups. On the other hand, the locus at 12p11.21 increases T1D risk *only* in families *without* MVCs, and only in the presence of a specific HLA allele. The 12p11.21 region shows no evidence of co-segregation with MVC (in **3/{3,X}** families) but does show significant evidence of co-segregation with T1D once the negatively contributing MVCs families are removed. The 22q11.21 signal supports the presence of a locus that alters the susceptibility to T1D, depending on the presence (or not) of MVCs. This region may contain a second locus that, alone, controls MVC risk. The 8q21.3 locus also appears to harbor a gene that confers susceptibility for MVC.

Stratification of the phenotype groups by HLA genotype indicated that HLA alleles contributed to heterogeneity through HLA genotype-dependent interaction with the loci we identified. In four out of the five significant linkage regions (the exception being the 1p36.12 peak), the strongest evidence for linkage was seen within specific *DRB1* allele strata. For loci linked to **MVCs** (1q32.1 and 8q21.3), strata enriched for the DRB1*03:*01* allele produce stronger linkage evidence, indicating that this allele may increase MVC risk in the presence of *certain genotypes* at other loci.

In general, regions showing linkage to MVCs also showed reduced heterogeneity compared to regions linked to **T1D** (LODs and α’s shown in Table 2 and supporting table in S1 Table) indicating that genes responsible for MVCs are specific and limited in number, and therefore easy to detect. Conversely, the fact that fewer T1D loci are seen within the **No complications** group (the larger sub group) may be the result of heterogeneity within T1D. Heterogeneity is probably the worst confounder for linkage, because evidence for one sub-form may reduce or even completely obliterate evidence for another sub-form, leading to weak linkage signals. For instance, the locus in the 1p36.12 region contributing to T1D expression becomes detectable only when heterogeneity is taken into account.

### Replication of previously reported T1D and MVC loci

The 1p36.12 region was previously reported as linked to diabetic retinopathy [8], but we found that this locus confers similar risk of T1D without MVCs and T1D with MVCs, suggesting that this locus has a general effect on T1D and is not an agent of heterogeneity. This region spans genes with plausible roles in the development of diabetic retinopathy, (e.g. *PADI1*, *PADI2*, *PADI3*, *and PADI4* [8], as well as genes known to be involved in kidney function (*CLCNKA* and *CLCNKB*). Therefore, we hypothesize that a locus in this region may be contributing to more than one type of MVC. Earlier linkage studies of T1D did not report these loci, perhaps because: a) they appear to be connected more with MVC expression than with T1D itself and b) they are detectable only when interaction with the HLA region is accounted for.

The 1q32.1 locus encompasses the *IL10* gene, which codes for an anti-inflammatory cytokine, interleukin 10 and has been reported by previously published T1D studies using genome-wide association and meta-analysis [9, 10].

Several regions of suggestive linkage near previously identified T1D susceptibility loci were also observed (see supporting Table in S2 Table), and one region associated with MVCs. Notable T1D loci within the suggestive linkage regions include the *CTLA4* gene, responsible for down-regulation of immune response [11–13], the *INS* gene, and the *GLIS3* gene, which is reported to play a role in beta cell apoptosis [10].

Combining MVCs into a single phenotype, may have reduced precision in determining influences on specific MVC phenotypes, such as those we identified in our previous work [3, 4]. However, we found sufficient evidence to support the idea that some T1D patients are genetically more susceptible to MVCs than others. If such genes are identified, both prognosis and care will be improved and we will gain substantial understanding of the causes of MVCs and how their expression is influenced by HLA type.

In conclusion, our study shows that T1D is an oligogenic and heterogeneous disease. There are indications of loci that are unique to either pedigrees that manifest MVCs or those that do not, but also of genetic loci common to the two groups. Some linked regions segregate only with T1D or MVC while others segregate with both T1D and MVC. There is clear evidence of epistatic interaction with the HLA region, and the DRB1*03:*01* allele is most often the effect modifier. Our results replicate earlier studies, while also nominating new loci responsible for T1D and MVC that would remain hidden if heterogeneity were unaccounted for. Where neither the MVC-based subgrouping, nor the *DRB1* genotype based stratification failed to account for heterogeneity, a deeper exploration of pedigree level characteristics such as sex, age of onset, and the presence of other HLA alleles needs to be conducted. Future studies should include investigation of individual MVCs.

## Research design and methods

### Subjects and sample assessment

Our dataset consisted of 415 families and 2,462 individuals (1,259 male, 1,203 female, and 896 with T1D) with diagnostic criteria and assessment protocols described previously [4]. Each family was ascertained through the presence of at least one member affected with T1D, i.e. the proband. Included in this study are the proband, parents, and one or more siblings. 395 out of the 415 families are multiplex for T1D, i.e. they contain at least one T1D-affected sibling of the proband. There are also 19 extended pedigrees that are multigenerational for T1D.

T1D case status required onset of T1D before age 30. The rate of misclassifying a T2D case as T1D was tested and found to be under 5% in our earlier study [4]. Probands and their family members were scored for three T1D-related MVCs: retinopathy, nephropathy and neuropathy. A T1D subject was considered “unaffected” for MVCs only if that individual was MVC-free for at least 15 years after T1D onset. There were 259 subjects with at least one MVC, belonging to 161 families. For this study, the presence of any MVC in a T1D-affected family member led us to assign the family to the “**Complications**” category. All analyses were based on the family classification “**Complications**” or “**No complications**”.

### Definition of phenotypes based on T1D and MVCs

In this study, our goal was to determine if families that have both T1D and MVCs are genetically different from families in which T1D subjects develop no MVCs. To detect differences, multiple phenotypes were defined based on both T1D and MVC status; pedigrees for linkage analysis were then grouped according to the phenotypes present in the family. The pedigree grouping procedure and definition of subjects’ affectedness status for the phenotypes analyzed are described in greater detail below.

1. **T1D group**: All families are included in the T1D group. Subjects with T1D are classified as “affected” and those without T1D as “unaffected”, ignoring their MVC status. Analyses of this group will detect loci that contribute to T1D regardless of MVC expression.
2. **Complications group**: Any family with at least one individual who has a T1D-related MVC is included in the **Complications** group. We emphasize that all subjects with MVCs will, by definition, also have T1D, but in this group, only subjects with MVCs are defined as “affected” for the analysis. Similarly, subjects with T1D but without MVCs are assigned the “unaffected” status, and individuals without T1D the “unknown” status in the **Complications** group.
3. **No complications group**: A family in which *no* member with T1D has any MVC is assigned to the **No complications** group. Individuals with T1D are assigned “affected” status and those without T1D the “unknown” status. Only non-MVC families with at least two T1D cases will contribute to the linkage signal.

In our data, many patients had more than one MVC, as seen in Fig 2A. Retinopathy was frequently observed on its own (128 cases), but patients with other MVCs, namely nephropathy and neuropathy, frequently also had retinopathy (93 cases). Individual MVCs were treated as a single phenotype, i.e., not analyzed separately, for three reasons. First, the overlap suggests that a common underlying pathology likely predisposes to all MVCs. Secondly there would be classification ambiguity. Third, and most important, our goal here was to see if there was evidence of heterogeneity *within* T1D based on the presence or absence of MVCs as a group.

### Stratification of phenotype groups by *DRB1* allele status

We looked for an HLA interaction with loci we identified by stratifying the pedigrees in each analysis on the basis of whether the proband’s genotype contained the two most frequent *HLA-DRB1* alleles observed within all probands, DRB1*03:*01* (33%) and DRB1*04:*01* (25%), following the design of our previous linkage studies [4–6]. The purpose of stratifying on HLA type is to detect epistatic (gene-gene) interaction between putative disease-causing loci and *HLA-DRB1*. In this report, DRB1*03:*01* is referred to as the **3** allele, DRB1*04:*01* as the **4** allele, and all other *DRB1* alleles as **X**.

For each phenotypic family group (previous section), six strata were created by partitioning the pedigrees as follows (1) 3/*: pedigrees whose probands have at least one **3** allele, (2) 4/*: probands have at least one **4** allele, (3) **3**/**{3,X}**: probands have one or two **3** alleles, but no **4** allele, (4) **4/{4,X}**: probands have one or two **4** allele(s) but no **3** allele, (5) **3/4**: proband genotypes are **3**/**4**, and (6) **X/X**: probands have neither the **3** nor the **4** allele. Sample sizes for each phenotype subset and its proband’s *HLA-DRB1* allele-based strata, including the number of pedigrees, individuals, and affected individuals, are shown in supporting Table in S3 Table. The frequencies of the **3** and **4** alleles within each subset are provided in columns 6 and 7 of this table.

### Genetic data

Genotypes for 6,700 autosomal SNPs were available for analysis. The average spacing between SNPs with respect to their genetic map positions is 0.5 centiMorgans (cM). Genotyping was carried out at the Center for Inherited Disease Research (CIDR) at the National Human Genome Research Institute. Here, we report results for chromosomes 1–5, and 7–22. Analysis of chromosome 6 in these families has been published previously [4].

### Statistical methods

Evidence for linkage of genomic regions to the phenotypes was evaluated using the maximized maximum LOD score (MMLS or MOD) method, allowing for heterogeneity [14]. Multipoint LOD (logarithm of odds) scores under the assumption of genetic heterogeneity were calculated assuming (i) a simple dominant model and (ii) a simple recessive model, both assuming a penetrance of 50%. The heterogeneity LOD (HLOD) score maximizes the score with respect to both the recombination fraction and the proportion of the pedigrees that are linked to a marker. The maximum of the HLODs from the two inheritance models (dominant and recessive)–MMLS—was used as the linkage statistic for each genetic position evaluated. The MMLS method has been shown to be more powerful than model-free methods [15], even when the exact mode of inheritance is unknown and when genetic heterogeneity is present. HLOD and MMLS were calculated using the GeneHunter program [16] under dominant and recessive models. Disease allele frequencies were set to 0.006 and 0.1 for the dominant and recessive models respectively. As in the previous study by Lipner et al. [4], disease model parameter values were selected as being optimal for detecting linkage. Those parameters differ from the Lipner et al. study because the current study examined the whole-genome and excluded chromosome 6, with the HLA region. In the previous study, all analyses initially assumed both dominant and recessive inheritance and the assumed model yielding the highest LOD score was then used for subsequent analyses at the identified loci. Most important, however, is the interpretation of these results with regards to the presence of heterogeneity is not affected. To test the robustness of our conclusions, we re-analyzed a subset of the genome-wide linkage scans using the higher parameter values used by Lipner et al., and noticed little difference in the peak MMLSs, (results not shown).

### Threshold for significance and interpretation of linkage results

We selected MMLS values of 3.0 and 2.0 as thresholds for genome-wide significant and genome-wide suggestive linkage, which correspond approximately to theoretical values used in LOD score analysis [17]. Although theoretical MMLS significance thresholds are higher [18], simulation studies have shown that these thresholds are adequate for analyzing small sibships such as ours [19]. Positive evidence of heterogeneity is noted where MMLS increases as a result of subgrouping by phenotype, and/or stratification by the probands’ *DRB1* genotype. Heterogeneity detected as a result of the *DRB1* genotype-based stratification is considered evidence of epistatic interaction.

Statistical substantiation for a difference between phenotypic subgroups’ linkage results was provided by the pre-divided sample test, which tests whether subgrouping substantially increases the linkage results [7, 20]. The difference of the total MMLSs from the sub groups, and MMLS of the combined sample is used to derive a p-value from a chi-square distribution, as shown in the equation below. Here, Z_i_ refers to the peak MMLS of the *ith* subgroup, and Z the peak MMLS of the undivided sample within a genomic interval. The LHS X^2^(i-1) is distributed as a chi-square with i-1 degrees of freedom. In this study, **Complications** and **No complications** constitute the subgroups, **T1D** the undivided sample, and the LHS is distributed as a chi-square with one degree of freedom. The pre-divided sample test is meaningful only if the total linkage score of the subgroups is greater than that of the T1D group. A p-value threshold of 0.05 was used to judge significance of the pre-divided sample test, corresponding to the upper 95^th^ percentile of the distribution of the chi-square statistic.

$${\text{X}}^{2}\left(\text{i}-1\right)=2\times \text{ln}\left(10\right)\left[{\text{Z}}_{\text{i}}-\text{Z}\right]$$ (1)

## Supporting information

S1 Table MMLS in all phenotype groups and strata corresponding to significant linkage regions. (DOCX)

S2 Table Linkage signals concurrent with published loci and MMLS ≥ 2.0. Note. Genes selected from review article by Barrett et al. 2009.
(DOCX)

S3 Table Sample size and allele frequency of alleles 3 and 4 within *DRB1*-based strata. Note. For each phenotype group, strata are ordered by the number of pedigrees. Affection status in No complications based on T1D affection status, affection status in Complications based on both T1D and MVC, individuals unaffected for T1D are assigned an unknown affection status. Allele frequencies shown in columns 6 and 7 were calculated using all genotyped individuals.
(DOCX)
