# Supplementary material for: Identifying genetic risk loci for diabetic complications and showing evidence for heterogeneity of type 1 diabetes based on complications risk
Source: PLoS One. 2018 Feb 14;13(2):e0192696. doi: 10.1371/journal.pone.0192696 (PMC5812614; doi:10.1371/journal.pone.0192696)
Supplement: S1 Table — (DOCX) [file pone.0192696.s001.docx]

MMLS in all phenotype groups and strata corresponding to significant linkage regions

| **Position (cM)** | | **Phenotype; DRB1 Stratum** | **MMLS** | | | | **LOD** | | | **Linked %** | |
| --- | --- | --- | --- | --- | --- | --- | --- | --- | --- | --- | --- |
| **Chromosome 1p 36.12** | | | | | | | | | | | |
| 49 | | T1D; Unstratified | 3.94 | | | | -89.70 | | | 17% | |
| 50 | | T1D; 3/* | 3.27 | | | | -48.16 | | | 20% | |
| 50 | | T1D; 3/{3,X} | 2.56 | | | | -26.74 | | | 24% | |
| 48 | | T1D; 4/* | 1.94 | | | | -42.77 | | | 17% | |
| 47 | | T1D; 3/4 | 1.11 | | | | -22.21 | | | 17% | |
| 48 | | T1D; 4/{4,X} | 0.91 | | | | -19.46 | | | 18% | |
| 49 | | T1D; X/X | 0.06 | | | | -18.64 | | | 5% | |
| 45 | | Complications; 3/{3,X} | 2.29 | | | | 1.92 | | | 72% | |
| 45 | | Complications; 3/* | 1.97 | | | | -0.98 | | | 46% | |
| 45 | | Complications; Unstratified | 1.23 | | | | -9.82 | | | 26% | |
| 45 | | Complications; 3/4 | 0.27 | | | | -2.90 | | | 21% | |
| 45 | | Complications; 4/* | 0.12 | | | | -7.81 | | | 9% | |
| 44 | | Complications; X/X | 0.07 | | | | -3.88 | | | 13% | |
| 53 | | Complications; 4/{4,X} | 0.00 | | | | -6.16 | | | 0% | |
| 49 | | No complications; Unstratified | 1.42 | | | | -59.31 | | | 13% | |
| 48 | | No complications; 4/{4,X} | 1.22 | | | | -1.04 | | | 52% | |
| 49 | | No complications; 3/{3,X} | 0.90 | | | | -19.66 | | | 18% | |
| 49 | | No complications; 3/* | 0.84 | | | | -38.83 | | | 13% | |
| 51 | | No complications; 4/* | 0.53 | | | | -10.39 | | | 23% | |
| 49 | | No complications; X/X | 0.34 | | | | -8.84 | | | 17% | |
| 44 | | No complications; 3/4 | 0.08 | | | | -19.27 | | | 6% | |
| **Chromosome 1q32.1** | | | | | | | | | | | |
| 206 | | T1D; 3/4 | | 1.27 | | -4.33 | | | 39% | | |
| 206 | | T1D; 4/* | | 0.61 | | -20.03 | | | 18% | | |
| 206 | | T1D; Unstratified | | 0.17 | | -56.72 | | | 7% | | |
| 206 | | T1D; 3/* | | 0.13 | | -33.26 | | | 7% | | |
| 210 | | T1D; X/X | | 0.11 | | -9.03 | | | 13% | | |
| 215 | | T1D; 4/{4,X} | | 0.07 | | -30.29 | | | 5% | | |
| 215 | | T1D; 3/{3,X} | | 0.00 | | -54.70 | | | 2% | | |
| 211 | | Complications; 3/{3,X} | | 3.02 | | 2.79 | | | 89% | | |
| 208 | | Complications; 3/* | | 1.73 | | -0.53 | | | 58% | | |
| 209 | | Complications; Unstratified | | 1.14 | | -5.10 | | | 39% | | |
| 214 | | Complications; X/Y | | 0.73 | | 0.46 | | | 74% | | |
| 205 | | Complications; 4/* | | 0.00 | | -11.32 | | | 0% | | |
| 214 | | Complications; 3/4 | | 0.00 | | -6.15 | | | 0% | | |
| 206 | | Complications; 4/{4,X} | | 0.00 | | -6.04 | | | 0% | | |
| 206 | | No complications; 3/4 | | 1.08 | | -1.33 | | | 47% | | |
| 206 | | No complications; 4/* | | 0.68 | | -11.26 | | | 24% | | |
| 206 | | No complications; Unstratified | | 0.24 | | -32.26 | | | 10% | | |
| 211 | | No complications; 4/{4,X} | | 0.23 | | -16.41 | | | 11% | | |
| 206 | | No complications; 3/* | | 0.16 | | -19.23 | | | 10% | | |
| 208 | | No complications; X/X | | 0.09 | | -4.61 | | | 17% | | |
| 215 | | No complications; 3/{3,X} | | 0.04 | | -32.80 | | | 4% | | |
| **Chromosome 8q21.3** | | | | | | | | | | | |
| 94 | | T1D; 3/{3,X} | | 2.31 | | -35.81 | | | 16% | | |
| 100 | | T1D; 3/* | | 1.53 | | -68.83 | | | 10% | | |
| 95 | | T1D; Unstratified | | 0.63 | | -136.82 | | | 4% | | |
| 91 | | T1D; 4/{4,X} | | 0.09 | | -14.28 | | | 10% | | |
| 91 | | T1D; 4/* | | 0.05 | | -29.88 | | | 5% | | |
| 97 | | T1D; 3/4 | | 0.00 | | -17.67 | | | 1% | | |
| 91 | | T1D; X/X | | 0.00 | | -24.49 | | | 0% | | |
| 100 | | Complications; 3/{3,X} | | 2.88 | | 2.02 | | | 64% | | |
| 91 | | Complications; Unstratified | | 2.49 | | -10.38 | | | 31% | | |
| 100 | | Complications; 3/* | | 2.17 | | -5.17 | | | 33% | | |
| 91 | | Complications; 4/{4,X} | | 0.95 | | -0.54 | | | 49% | | |
| 91 | | Complications; 4/* | | 0.66 | | -6.60 | | | 25% | | |
| 91 | | Complications; X/X | | 0.20 | | -0.79 | | | 43% | | |
| 95 | | Complications; 3/4 | | 0.00 | | -5.10 | | | 0% | | |
| 91 | | No complications; 3/* | | 0.10 | | -22.41 | | | 8% | | |
| 94 | | No complications; 3/{3,X} | | 0.09 | | -14.01 | | | 10% | | |
| 91 | | No complications; 3/4 | | 0.02 | | -8.81 | | | 5% | | |
| 91 | | No complications; 4/* | | 0.01 | | -16.70 | | | 2% | | |
| 91 | | No complications; Unstratified | | 0.00 | | -40.10 | | | 1% | | |
| 91 | | No complications; X/X | | 0.00 | | -15.44 | | | 0% | | |
| 91 | | No complications; 4/{4,X} | | 0.00 | | -19.79 | | | 0% | | |
| **Chromosome 12p11.21** | | | | | | | | | | | |
| 61 | | T1D; X/X | | 1.66 | | -11.37 | | | 26% | | |
| 62 | | T1D; Unstratified | | 0.36 | | -69.55 | | | 9% | | |
| 62 | | T1D; 3/* | | 0.05 | | -45.11 | | | 4% | | |
| 62 | | T1D; 3/{3,X} | | 0.04 | | -28.12 | | | 6% | | |
| 56 | | T1D; 4/{4,X} | | 0.03 | | -33.40 | | | 3% | | |
| 62 | | T1D; 3/4 | | 0.01 | | -17.40 | | | 3% | | |
| 62 | | T1D; 4/* | | 0.00 | | -74.64 | | | 0% | | |
| 54 | | Complications; Unstratified | | 0.36 | | -8.46 | | | 25% | | |
| 54 | | Complications; 4/{4,X} | | 0.28 | | -1.35 | | | 41% | | |
| 54 | | Complications; 4/* | | 0.28 | | -3.94 | | | 28% | | |
| 55 | | Complications; 3/{3,X} | | 0.20 | | -3.62 | | | 21% | | |
| 54 | | Complications; 3/* | | 0.17 | | -7.35 | | | 13% | | |
| 55 | | Complications; 3/4 | | 0.05 | | -2.68 | | | 18% | | |
| 54 | | Complications; X/X | | 0.02 | | -1.54 | | | 13% | | |
| 57 | | No complications; X/X | | 3.21 | | -0.09 | | | 52% | | |
| 62 | | No complications; Unstratified | | 0.28 | | -40.72 | | | 10% | | |
| 62 | | No complications; 3/4 | | 0.01 | | -11.07 | | | 4% | | |
| 61 | | No complications; 4/{4,X} | | 0.01 | | -20.59 | | | 1% | | |
| 52 | | No complications; 3/{3,X} | | 0.00 | | -38.15 | | | 1% | | |
| 52 | | No complications; 4/* | | 0.00 | | -50.98 | | | 0% | | |
| 52 | | No complications; 3/* | | 0.00 | | -67.88 | | | 0% | | |
| **Chromosome 22 q11.21** | | | | | | | | | | | |
| 12 | | T1D; 3/4 | | 3.24 | | -1.39 | | | 56% | | |
| 12 | | T1D; 3/* | | 1.96 | | -22.08 | | | 28% | | |
| 9 | | T1D; 4/* | | 1.94 | | -18.04 | | | 31% | | |
| 9 | | T1D; Unstratified | | 1.24 | | -52.53 | | | 17% | | |
| 7 | | T1D; 4/{4,X} | | 0.27 | | -14.24 | | | 17% | | |
| 8 | | T1D; 3/{3,X} | | 0.17 | | -22.08 | | | 11% | | |
| 7 | | T1D; X/X | | 0.00 | | -24.15 | | | 0% | | |
| 17 | | Complications; 3/4 | | 2.22 | | 2.22 | | | 100% | | |
| 12 | | Complications; 4/* | | 0.95 | | -2.53 | | | 36% | | |
| 17 | | Complications; 3/* | | 0.29 | | -6.14 | | | 18% | | |
| 8 | | Complications; 4/{4,X} | | 0.13 | | -3.16 | | | 18% | | |
| 11 | | Complications; Unstratified | | 0.04 | | -17.85 | | | 6% | | |
| 7 | | Complications; X/X | | 0.00 | | -6.29 | | | 0% | | |
| 14 | | Complications; 3/{3,X} | | 0.00 | | -8.22 | | | 0% | | |
| 13 | | No complications; 3/* | | 1.71 | | -10.94 | | | 33% | | |
| 8 | | No complications; Unstratified | | 1.54 | | -27.19 | | | 24% | | |
| 7 | | No complications; 4/* | | 1.36 | | -11.28 | | | 32% | | |
| 11 | | No complications; 3/4 | | 1.33 | | -4.00 | | | 43% | | |
| 14 | | No complications; 3/{3,X} | | 0.63 | | -6.50 | | | 27% | | |
| 7 | | No complications; 4/{4,X} | | 0.34 | | -6.56 | | | 24% | | |
| 14 | | No complications; X/X | | 0.09 | | -4.31 | | | 16% | | |
