# Supplementary material for: Identifying genetic risk loci for diabetic complications and showing evidence for heterogeneity of type 1 diabetes based on complications risk
Source: PLoS One. 2018 Feb 14;13(2):e0192696. doi: 10.1371/journal.pone.0192696 (PMC5812614; doi:10.1371/journal.pone.0192696)
Supplement: S2 Table — Note. Genes selected from review article by Barrett et al. 2009. (DOCX) [file pone.0192696.s002.docx]

Linkage signals concurrent with published loci and MMLS ≥ 2.0

| **Location** | **Phenotype;**  **DRB1 stratum** | **MMLS; LOD; α(%)** | **Gene** |
| --- | --- | --- | --- |
| 1p36.12 | T1D; unstratified | 3.94; -90; 17 | *PADI1, PADI3, PADI4* |
| 1q32.1 | Complications; 3/{3,X} | 3.02; 2.79; 89 | *IL10* |
| 2q33.1 | No Complications; 4/{4,X} | 2.32; -0.52; 58 | *CTLA4* |
| 9p24.2 | T1D; 3/4 | 2.84; -0.87; 54 | *GLIS3* |
| 11p15.4 | T1D; 4/{4,X} | 2.07; -11; 35 | *INS* |
| 12q14.3 | No Complications; unstratified | 2.02; -25.47; 28 | *CYP27B1* |
| 14q32.2 | Complications; 3/4 | 2.00; 1.32; 65 | *DLK1, RAGE* |

Note. Genes selected from review article by Barrett et al. 2009
