# Supplementary material for: Identifying genetic risk loci for diabetic complications and showing evidence for heterogeneity of type 1 diabetes based on complications risk
Source: PLoS One. 2018 Feb 14;13(2):e0192696. doi: 10.1371/journal.pone.0192696 (PMC5812614; doi:10.1371/journal.pone.0192696)
Supplement: S3 Table — Note. For each phenotype group, strata are ordered by the number of pedigrees. Affection status in No complications based on T1D affection status, affection status in Complications based on both T1D and MVC, individuals unaffected for T1D are assigned an unknown affection status. Allele frequencies shown in columns 6 and 7 were calculated using all genotyped individuals. (DOCX) [file pone.0192696.s003.docx]

Sample size and allele frequency of alleles **3** and **4** within DRB1-based strata

| **DRB1 stratum** | **Number of Pedigrees** | **Individuals** | **Affected; Unaffected^2^** | **Genotyped** | **Frequency of allele 3** | **Frequency of allele 4** |
| --- | --- | --- | --- | --- | --- | --- |
| **T1D** | | | | |  |  |
| 3/* | 250 | 1,504 | 533; 967 | 1,211 | 0.35 | 0.15 |
| 4/* | 200 | 1,149 | 422; 719 | 946 | 0.17 | 0.35 |
| 3/{3,X} | 154 | 950 | 336; 611 | 754 | 0.37 | 0.04 |
| 4/{4,X} | 104 | 595 | 225; 363 | 489 | 0.04 | 0.37 |
| 3/4 | 96 | 554 | 197; 356 | 457 | 0.31 | 0.33 |
| X/X | 61 | 363 | 138; 220 | 295 | 0.07 | 0.07 |
| **Complications** | | | | |  |  |
| 3/* | 83 | 552 | 134; 56 | 408 | 0.47 | 0.17 |
| 4/* | 74 | 432 | 109; 51 | 360 | 0.17 | 0.52 |
| 3/{3,X} | 57 | 393 | 91; 47 | 270 | 0.49 | 0.03 |
| 4/{4,X} | 46 | 262 | 64; 38 | 222 | 0.02 | 0.53 |
| 3/4 | 28 | 170 | 45; 13 | 138 | 0.42 | 0.50 |
| X/X | 28 | 190 | 48; 28 | 131 | 0.09 | 0.11 |
| **No complications** | | | | |  |  |
| 3/* | 165 | 943 | 338; 602 | 794 | 0.36 | 0.16 |
| 4/* | 126 | 717 | 262; 449 | 586 | 0.18 | 0.35 |
| 3/{3,X} | 97 | 559 | 199; 358 | 475 | 0.40 | 0.04 |
| 4/{4,X} | 68 | 384 | 139; 244 | 319 | 0.31 | 0.33 |
| 3/4 | 58 | 333 | 123; 205 | 267 | 0.04 | 0.37 |
| X/X | 36 | 202 | 77; 123 | 164 | 0.05 | 0.05 |

Note. For each phenotype group, strata are ordered by the number of pedigrees. Affection status in **No** **complications** based on T1D affection status, affection status in **Complications** based on both T1D and MVC, individuals unaffected for T1D are assigned an unknown affection status. Allele frequencies shown in columns 6 and 7 were calculated using all genotyped individuals.
